# Use of insecticide treated net and malaria preventive education: effect on malaria parasitemia among people living with AIDS in Nigeria, a cross-sectional study

**DOI:** 10.1186/1447-056X-12-2

**Published:** 2013-06-22

**Authors:** Samuel Anu Olowookere, Najemdeen Ajao Adeleke, Emmanuel Akintunde Abioye-Kuteyi, Ijeoma Soromtochi Mbakwe

**Affiliations:** 1 Department of Community Health, College of Health Sciences, Obafemi Awolowo University, Ile-Ife, Osun State, Nigeria; 2 Department of Obstetrics and Gynaecology, Osun State University, Osogbo, Osun state, Nigeria; 3 Antiretroviral clinic, State hospital, Osogbo, Osun state, Nigeria

**Keywords:** Malaria, PLWHA, ITN use, Preventive education

## Abstract

**Background:**

Malaria and HIV are major causes of morbidity and mortality in sub-Saharan Africa with both diseases highly endemic in Nigeria. This study was conducted to assess the effect of long lasting insecticide treated net (ITN) use and malaria preventive education on burden of malaria parasite among people living with AIDS (PLWHA) at Osogbo southwestern Nigeria.

**Method:**

A descriptive cross-sectional study of newly recruited consenting PLWHA that were screened consecutively for malaria, those positive were treated with artemisinin combination therapy. All PLWHA were educated about malaria infection, given ITN and followed up monthly for three months when they were rescreened for malaria infection. Data collected was analyzed using descriptive and inferential statistics.

**Result:**

A total of 392 (92%) PLWHA completed the study. Mean age of the respondents was 33 ± 11.6 years. They were 120 (31%) males and 272 (69%) females. Majority (80%) were married, over 33% completed secondary education while 21% had tertiary education. Most were traders (40%) and artisans (25%). About 60% had Plasmodium falciparum malaria parasitemia at baseline which drastically reduced to 5% at three months with ITN use and malaria prevention education.

**Conclusion:**

Malaria is a major preventable condition among PLWHA. Preventive education and ITN use reduced malaria parasite burden among this population.

## Background

Malaria and HIV/AIDS are leading causes of morbidity and mortality in sub-Saharan Africa [1]. Both diseases are highly endemic in Nigeria [2]. Malaria is the most common life threatening infection with one million deaths per year, 300–500 million infections per year, about 90% of the death occurring in sub-Saharan Africa [3]. Most victims were children less than 5 years of age and pregnant women [3]. Human malaria is a parasitic infection caused by *Plasmodium falciparum, Plasmodium vivax, Plasmodium ovale* and *Plasmodium malariae*[4]. Of these four species of plasmodium, *Plasmodium falciparum* causes the most severe malaria illness and death throughout the world. *Plasmodium falciparum* is known to be the most devastating in Nigeria [2]. Malaria is transmitted through the bite of an infected female anopheles mosquito [4].

Nigeria accounts for a quarter of all malaria cases in the World Health Organization African Region [2]. It is endemic throughout the country with more than 90% of the total population at risk of stable endemic malaria. At least 50% of the population suffers from at least one episode of malaria each year [4]. Transmission in the south occurs all-year round, and is more seasonal in the north [4]. Almost all cases are caused by *P. falciparum* but most are unconfirmed [4]. There is no evidence of a systematic decline in malaria burden; the upward trend in numbers of cases and deaths is probably due to improvements in reporting [4].

HIV infection increases the risk and severity of malaria [3]. The population-level impact depends on HIV prevalence, the age distribution of both infections which for malaria is determined by the transmission intensity, and their geographic overlap [5]. Local distributions of CD4 cell counts and clinical stages of HIV-infected patients are also important because the effects of HIV multiply with increasing immunosuppression [3]. Studies on adults in areas of high malaria transmission showed that HIV-1 infection increased case fatality among hospitalized persons with severe malaria by 1.6- to 2.5- fold, while the incidence of severe malaria, a precursor of fatal episodes, increased by 2.7-fold. HIV-1 increases malaria deaths by increasing the proportion of severe cases, case fatality among them, and the failure rate of antimalarials treatment. HIV-1 increased malaria incidence by 0.20% to 28% across countries. HIV-1 increased malaria deaths by 0.65% to 114% across countries [5]. In HIV-infected adults, pregnant women, and children, malaria is among the simplest infections to prevent and treat [6].

The malaria preventive health behaviour had been found to be generally poor across Nigeria with less than 10% respondents using long lasting insecticide treated net (ITN) [2]. Malaria is a major public health problem in Nigeria, accounting for about 60% of all outpatient attendances and 30% of all hospital admissions [4]. It is estimated that malaria is responsible for nearly 110 million clinical cases and an estimated 300,000 deaths per year, including up to 11% of maternal mortality [2].

This study was conducted to assess the effect of ITN use and malaria preventive education on burden of malaria parasitemia among people living with AIDS (PLWHA) at Osogbo southwestern Nigeria.

## Methodology

### Study site

The antiretroviral (ARV) clinic located at State hospital Osogbo, Osun State, southwest Nigeria was established February 2009. The hospital is a free treatment General hospital attending to about 10,000 patients weekly. The ARV Clinic offers comprehensive HIV/AIDS care for all the 31 local government health facilities in Osun State and beyond. This clinic is supported by the Osun State Ministry of Health in collaboration with Global HIV/AIDS Initiative in Nigeria. PLWHA receive care and support including antiretroviral therapy at the clinic which opens every Mondays and Wednesdays from 8 am to 4 pm. It attends to 100–140 PLWHA weekly. Patients on care were usually routinely followed up monthly while very ill patients were admitted into the hospital medical wards.

### Study design

This is a descriptive cross-sectional study from June to December 2010. The study population included new PLWHA recruited consecutively by trained research assistants after they have consented to participate following through explanation of the study by the researchers to them and/or their family caregivers (where respondent was less than 18 years). The required sample size of 334 was calculated using an appropriate statistical formula for estimating the minimum sample size in descriptive health studies [n = Z^2^pq/d^2^] [7] and finding from a previous study [6] where 32% of PLWHA studied had malaria. The minimum sample size was increased by 10% to take care of incomplete/non response and refusals. The informed consent form and a pretested questionnaire that included information on biodata, awareness of malaria prevention, and previous ITN use were administered to the research participants and their family caregivers by the researchers at beginning of the study. These consenting PLWHA had their blood film screened consecutively for malaria parasite at the beginning of the study. The respondents that were malaria parasite positive were treated free with artemisinin combination therapy regimen while all clients’ and their family caregivers’ undergone education about malaria and HIV infection. They were then given ITN free which they were encouraged to sleep under every night. They were followed up monthly for three months when they had their blood film rescreened for malaria parasite. The study participants were also interviewed with these questionnaires during their monthly visits to the clinic. Also these respondents were visited at home by the antiretroviral clinic home visit team to ensure adherence to antiretroviral drug therapy, cotrimoxazole, preventive education and ITN use.

Ethical consideration included taking informed consent from respondents and their family caregiver, using serial numbers and not names to maintain confidentiality of information obtained. Permission to conduct the study was granted by the Osun State Hospital Ethics and Research Committee. Data collected were kept in a password computer.

Data obtained was entered into SPSS version 16. Summary statistics using mean, median, standard deviation, range for continuous variables and frequency/percentages for categorical variables were generated. Chi square statistics was used to test association between categorical variables with p value less than 0.05 taken as significant.

## Results

A total of 392 PLWHA out of 425 completed the study (92%). Thirty-three respondents opted out of the study for personal reasons such as unscheduled traveling. No respondent died or were lost to follow up during the study period. Mean age of the respondents was 33 years ± 11.6 years (Range = 13-60 years).

Table 1 reported the sociodemographic characteristics of respondents. They were 120 (31%) males and 272 (69%) females. Majority were married (80%), 33% completed secondary education while 21% had tertiary education. Most were traders (40%) and artisans (25%). At baseline all respondents saw malaria as a threat to good health while 42% were aware that malaria could be prevented but none sleep under ITN at home. Reasons for non use of ITN were non availability (54%), exorbitant price (48%), and ignorance (22%). Only 15% used insecticide spray or coil which they said was inadequate as they still get malaria despite constant use.

**Table 1:** Sociodemographic characteristics of PLWHA attending State hospital, Osogbo

**Sociodemographic characteristics**	**Frequency (N = 392)**	**%**
Age group (years)		
13–18	30	7.7
19–29	92	23.5
30–39	166	42.3
≥40	104	26.5
Sex		
Male	120	30.6
Female	272	69.4
Education		
None	90	23.0
Primary	88	22.4
Secondary	130	33.2
Tertiary	84	21.4
Marital status		
Single	64	16.3
Married	314	80.1
Divorced/widowed	14	3.6
Occupation		
Trading	158	40.3
Civil servant	56	14.3
Artisan	98	25.0
Police	18	4.6
Clergy	6	1.5
Unemployed	56	14.3

**Figure 1:**
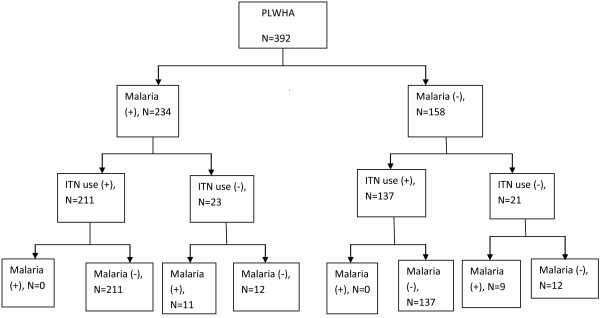
Flowchart of ITN use and malaria parasitemia among PLWHA.

Table 2 reported the pattern of malaria parasitemia and ITN use among the study participants. About 60% had Plasmodium falciparum malaria parasitemia at baseline which drastically reduced to 5% at three months with ITN use and malaria prevention education. Figure 1 showed that PLWHA using ITN had no malaria parasitemia at three months.

**Table 2:** Pattern of malaria parasitemia and ITN use among PLWHA at state hospital, Osogbo, Nigeria

**Malaria parasite result/ITN use**	**Frequency**	**%**
Result at baseline		
Positive	234	59.7
Negative	158	40.3
Result at three month		
Positive	20	5.1
Negative	372	94.9
ITN given at baseline		
Yes	392	100
ITN use at 3 months		
Yes	348	88.8
No	44	11.2

Table 3 showed the respondent’s sociodemographic characteristics and malaria positivity at baseline and three months follow up. Majority (68%) of the respondents in the formal sector was malaria parasite positive at baseline but all were malaria parasite negative by third month. Over 45% of respondents who were not using ITN had malaria parasitemia at third month follow up.

**Table 3:** Respondent’s sociodemographic characteristics and malaria positivity at baseline and three months follow up

**Characteristics**	**Malaria parasite (baseline)**		**p value**	**Malaria parasite (3rd month)**		**p value**
	**Positive (%)**	**Negative (%)**		**Positive (%)**	**Negative (%)**	
Age group						1.000
13–29	78 (63.9)	44 (36.1)	0.25	6 (4.9)	116 (95.1)	
≥30	156 (57.8)	114 (42.2)		14 (5.2)	256 (94.8)	
Sex						0.46
Male	66 (55)	54 (45)	0.21	8 (6.7)	112 (93.3)	
Female	168 (61.8)	104 (38.2)		12 (4.4)	260 (95.6)	
Education						0.25
≤Primary	114 (64)	64 (36)	0.11	12 (6.7)	166 (93.3)	
≥Secondary	120 (56.1)	94(43.9)		8 (3.7)	206 (96.3)	
Marital status						1.000
Single	46 (59)	32 (41)	0.89	4 (5.1)	74 (94.9)	
Married	188 (59.9)	126 (40.1)		16 (5.1)	298 (94.9)	
Occupation						0.019
Informal sector	184 (57.9)	134 (42.1)	0.13	20 (6.3)	298 (93.7)	
Formal sector	50 (67.6)	24 (32.4)		0 (0)	74 (100)	
ITN use						0.001
Yes	0 (0)	0 (0)		0 (0)	348 (100)	
No	234 (59.7)	158 (40.3)		20 (45.5)	24 (54.5)	

Common reasons for inconsistent or non use of ITN included night duty (39%), reaction to the permethrin embedded in the net (27%) while some respondents never liked sleeping under ITN (23%).

## Discussion

This study assessed the effect of ITN use and malaria preventive education on burden of malaria parasitemia among people living with AIDS. It reported that preventive education and ITN use reduced malaria parasitemia over a period of three months. The high malaria burden at baseline reduced remarkably by three months. Previous studies had shown this among pregnant women and children [2,4].

Majority of the study population had malaria at baseline. This finding is expected as the study area is an area of intense year round malaria transmission. Goselle et al. in 2009 in a study on malaria infection among PLWHA in Jos, Nigeria reported 32% while Saracino et al. in 2012 reported 26% in Beira, Mozambique [6,8]. However a much higher burden was reported in the present study because most PLWHA studied had no malaria preventive education and no access to ITN at baseline. Studies had shown that malaria if not treated and prevented will further reduced the quality of life of these immunocompromised population [3,5,6].

This study reported that ITN use protects against malaria among the participants. This entails that malaria preventive education and ITN should be routinely given to PLWHA to prevent malaria among them, thereby improving their quality of life.

Most respondents gave financial constraints and non availability as their reason for non use of ITN at baseline. Various studies on challenges to ITN use in this environment had reported this finding. For example, Olajide et al. in a study on challenges with ITN use among pregnant women reported that pregnant women did not sleep under ITN because of non availability (53%) and cost (24%) [9]. This implied that ITN must be made available and affordable or free to all PLWHA to reduce malaria burden among them.

This study further showed that consenting PLWHA should be routinely visited at home to observe the conditions they lived. This will improve relationship between these PLWHA and the healthcare team. Studies had showed that the living conditions of patients affect their health and that improving these living conditions will improve their quality of life [4,10].

This study also reported that the respondents working in the formal sector with high parasitemia at baseline had no malaria parasitemia after three months of continuous ITN use at night and malaria preventive education. This was due to strict adherence to instructions given by their healthcare workers. This could be explained by higher formal education and better environmental conditions of respondents working in the formal sector. Studies had reported the positive influence of formal education on adherence to malaria preventive messages [2,4].

Also this study found that more than two-fifth of respondents who were not using ITN had malaria parasitemia at third month follow up. This implies that ITN use prevent exposure to mosquito bite hence thwarting the plasmodium organism transmission. Various studies had reported that mosquito bite is necessary for the transmission of the plasmodium organism [11-13].

This study reported that the main reasons for inconsistent or non-use of ITN included night duty and reaction to permethrin embedded in the net. Permethrin impregnated ITN had been shown in various studies to prevent the plasmodium transmission however reaction to this chemical agent cannot be foreclosed [13,14]. Since the most important prevention against malaria is to avoid the infected bite from the mosquito which is only possible when the individual actually slept under a properly deployed net during the night [13,15]. Also the PLWHA on night duty could cover the exposed parts of their body to prevent exposure to mosquito bite during this period while people who never liked sleeping under the ITN need further malaria preventive education to see the merits of preventing exposure to the plasmodium organism through sleeping under the ITN every night.

Limitation to the study is that it is a preliminary study on effect of preventive education and ITN use on malaria parasitemia among PLWHA. Also, a convenience sample of PLWHA was selected.

## Conclusion

Preventive education and consistent ITN use reduced malaria infection among PLWHA. There should be ongoing preventive education on malaria with free provision of ITN with home visits by healthcare workers targeted at ensuring adherence to ITN use.

## Competing interest

The authors declare that they have no competing interests.

## Authors’ contributions

SAO and NAA made substantial contributions to conception and design of the study while all the authors were involved in data collection, analysis and interpretation. All authors were involved in writing the manuscript and approved the final copy.

## Authors’ information

SAO lectures at Department of Community Health, Faculty of Clinical Sciences, College of Health Sciences, Obafemi Awolowo University, Ile-Ife, Osun State, Nigeria. Areas of research interest include communicable and non communicable diseases, family issues; NAA lectures at Department of O&G, Osun State University, Osogbo, Nigeria. Areas of research interest include reproductive and infection epidemiology, gynaecological diseases; EAA is an associate professor at the Department of Community Health, Faculty of Clinical Sciences, College of Health Sciences, Obafemi Awolowo University, Ile-Ife, Osun State, Nigeria. Areas of research interest include communicable and non communicable diseases, family issues; ISM is a medical officer at the Antiretroviral clinic, State hospital, Osogbo, Nigeria.

Study site: Antiretroviral clinic, State hospital, Osogbo, Osun state, Nigeria
